# Measured Energy Expenditure Using Indirect Calorimetry in Post-Intensive Care Unit Hospitalized Survivors: A Comparison with Predictive Equations

**DOI:** 10.3390/nu14193981

**Published:** 2022-09-25

**Authors:** Anne-Françoise Rousseau, Marjorie Fadeur, Camille Colson, Benoit Misset

**Affiliations:** 1 Department of Intensive Care, University Hospital of Liège, University of Liège, 4000 Liège, Belgium; 2 Multidisciplinary Nutrition Team, University Hospital of Liège, 4000 Liège, Belgium

**Keywords:** nutrition, critical care, indirect calorimetry, resting energy expenditure, survivors

## Abstract

Actual energy needs after a stay in intensive care units (ICUs) are unknown. The aims of this observational study were to measure the energy expenditure (mEE) of ICU survivors during their post-ICU hospitalization period, and to compare this to the estimations of predictive equations (eEE). Survivors of an ICU stay ≥ 7 days were enrolled in the general ward during the first 7 days after ICU discharge. EE was measured using the Q-NRG calorimeter in canopy mode. This measure was compared to the estimated EE using the Harris–Benedict (HB) equation multiplied by a 1.3 stress factor, the Penn–State (PS) equation or the 30 kcal weight-based (WB) equation. A total of 55 adults were included (67.3% male, age 60 (52–67) y, body mass index 26.1 (22.2–29.7) kg/m^2^). Indirect calorimetry was performed 4 (3–6) d after an ICU stay of 12 (7–16) d. The mEE was 1682 (1328–1975) kcal/d, corresponding to 22.9 (19.1–24.2) kcal/kg/day. The eEE values derived using HB and WB equations were significantly higher than mEE: 3048 (1805–3332) and 2220 (1890–2640) kcal/d, respectively (both *p* < 0.001). There was no significant difference between mEE and eEE using the PS equation: 1589 (1443–1809) kcal/d (*p* = 0.145). The PS equation tended to underestimate mEE with a bias of −61.88 kcal and a wide 95% limit of agreement (−717.8 to 594 kcal). Using the PS equation, agreement within 15% of the mEE was found in 32/55 (58.2%) of the patients. In the present cohort of patients who survived a prolonged ICU stay, mEE was around 22–23 kcal/kg/day. In this post-ICU hospitalization context, none of the tested equations were accurate in predicting the EE measured by indirect calorimetry.

## 1. Introduction

After a stay in an intensive care unit (ICU), survivors are at high risk of malnutrition. Critical illness is associated with metabolic alterations as consequences of the systemic inflammation [1]. On the other hand, suboptimal nutritional intakes are frequent during ICU stays [2]. After ICU discharge, oral nutrition is the most common mode of nutrition provision [3]. However, this route exposes patients to the highest risk of nutritional deficiencies. Barriers to adequate nutritional intake in the post-ICU period are related to reduced appetite, taste changes, chewing difficulties, swallowing impairment or cognitive disorders, and inadequate food services. Some previous reports confirm this issue, describing inadequate intakes in patients on the oral route after mechanical ventilation liberation [4,5].

Malnutrition has systemic repercussions for muscle mass and function, the gastrointestinal tract, mental health and the endocrine system [6,7]. Disorders affecting these domains are part of the post-intensive care syndrome, known to be associated with a decrease in health-related quality of life and an increase in healthcare utilization [8]. Optimal nutritional care is thus fundamental to the recovery process. The existing literature on nutritional care in critically ill patients is mainly centered on the acute phase, and the optimal quantity and quality of energy and protein intakes are still poorly defined in ICU survivors. In the period of post-ICU hospitalization, higher energy and protein requirements are proffered in order to face persistent catabolism and secondary anabolism, thus preventing further muscle loss. According to experts, energy intakes could be increased to 125% of the predicted requirements, or to 30 kcal/kg/day, while protein intakes could reach 1.5–2 g/kg/day [9]. However, data on the energy expenditure and protein consumption in the post-ICU period are rare [10], and there are no recommendations guiding nutrition provision during this period.

Indirect calorimetry is the most accurate way to measure energy expenditure, based on the measurement of inspired and expired oxygen and carbon dioxide concentrations. This technique is now recommended as the gold standard to determine energy needs in the critical care setting [11,12]. This task has been made easier since the development of a new-generation indirect calorimeter, validated against the reference method for gas composition measurement (mass spectrometry), with the advantages of being accurate, reliable, user-friendly and financially affordable. Advantageously for the post-ICU condition, the measurement can be performed in spontaneously breathing patients, using a canopy hood [13,14,15].

The definition of energy targets specifically for ICU survivors is a crucial step prior to prescribing adequate nutrition therapy and thus delivering optimal nutritional care. The primary aim of the present observational study was to measure the energy expenditure of ICU survivors during their post-ICU hospitalization period using indirect calorimetry. The secondary aim was to compare the measured and estimated energy expenditures, based on common predictive equations.

## 2. Materials and Methods

### 2.1. Population

Over 8 months in 2021 and 2022, all consecutive critically ill survivors who were discharged from our ICU after a stay ≥ 7 days were recruited in the present observational study. Patients were enrolled in the general ward, during the first 7 days following ICU discharge. Oxygen therapy (either with face mask or nasal canula), confusion or agitation, isolation for COVID-19 infection or multi-drug-resistant bacteria, and refusal to participate were considered as exclusion criteria. Patients discharged from our burn ICU were not included in the present cohort.

The study was conducted according to the guidelines of the Declaration of Helsinki (1964) and its later amendments, after approval by the local ethics committee of our university hospital (Chairperson: Pr Vincent Seutin, National Ref B707201732960, Local Ref 2019/350, 14 May 2020) and registration on ClinicalTrials.gov (NCT04500483). Informed consent was obtained from patients before enrollment.

### 2.2. Indirect Calorimetry

The measurements were performed using the Q-NRG indirect calorimeter in canopy mode (Cosmed, Rome, Italy and Baxter, Deerfield, IL, USA). In canopy dilution mode, a digital turbine flowmeter operates in series with the internal blower to draw air at a constant flow rate through the canopy hood. The pumping rate is automatically calculated based on the patient’s weight and can be adjusted during the measurement to prevent too high or too low carbon dioxide (CO_2_) concentration in the hood. Inspired and expired air samples are collected in an internal micro-mixing chamber. These samples are then analyzed with a chemical fuel cell O_2_ sensor and a non-dispersive infrared adsorption digital CO_2_ sensor. Mean values of oxygen consumption (VO_2_) and carbon dioxide production (VCO_2_) are reported every 20 s. Energy expenditure (EE) is calculated according to the Weir equation. The respiratory quotient (RQ) is also calculated.

Gas analyzers were automatically calibrated against room air before each measurement. A calibration of the internal turbine flowmeter using a calibration syringe, and the calibration of the gas analyzers against a precision gas mixture, were performed monthly, as recommended by the manufacturer.

IC was performed in non-fasted patients, in a quiet environment with no sources of distraction. Patients lay on the bed for 10 min before starting the measurement. The measurement lasted at least 20 min, with the first 5 min used to establish a stable baseline. The measured EE was considered to be the resting energy expenditure, with limited physical activity observed during this post-ICU phase [16].

### 2.3. Estimated Energy Expenditure

Population-specific equations for EE estimation in post-ICU phase are nonexistent. The measured EE was compared to three estimated EEs, calculated using three predictive formulas (Table 1): the Harris–Benedict equation (raw result and a second result multiplicated by a stress factor of 1.3 [17]), the Penn–State equation in its modified version for spontaneously breathing patients [18], and the weight-based formula for ICU survivors (30 kcal/kg/day) according to practical guidance from experts in the field [9].

### 2.4. Other Clinical Data

Demographic data (age, sex, actual weight, height, body mass index (BMI)) were recorded. Actual weight was used for patients with BMI < 25 kg/m^2^. Ideal body weight (IBW) was considered as the expected weight for BMI 25 in overweight patients. In obese patients (BMI ≥ 30), the adjusted body weight was calculated as follows: IBW + 0.33 × (actual weight-IBW) [11]. Data about the feeding regimen and ICU stay were also recorded.

The biological data were generated from one single laboratory (Unilab, CHU de Liège, Liège, Belgium) accredited by the ISO 15,189 Guideline. Blood levels of total protein were assayed using turbidimetry (Alinity C, Abbott, IL, USA); the reference range was 58–83 g/L. Blood levels of albumin were assayed by spectrophotometry (Alinity C, Abbott, IL, USA). The reference ranges for ≤60 years were 35–52 and for >60 years, 32–46 g/L. Blood prealbumin and C-reactive protein concentrations were assayed using immunoturbidimetry (Alinity C, Abbott, IL, USA). The reference ranges were 0.2–0.4 g/L and 0–5 mg/L.

### 2.5. Statistical Analysis

Statistical analysis was performed using Graphpad Prism (version 6.0 for Mac OSX, Graphpad Inc., San Diego, CA, USA). Normality was assessed using the Shapiro–Wilk test. As some datasets did not pass the normality test, the results were expressed as medians with lower and upper quartiles (Q1–Q3) for quantitative parameters, or as counts and proportions for qualitative parameters. Comparisons between data were made using the Mann–Whitney test or the Wilcoxon matched-pairs signed rank test. Correlations between measured and estimated EE were tested using the Spearman test. Each formula was tested against the reference method (IC) for bias using Bland–Altman analysis for accuracy. Accuracy was defined as the percent of estimates falling within 85% and 115% of measured EE. A *p* value < 0.05 was considered statistically significant.

## 3. Results

### 3.1. Patients

**Figure 1:**
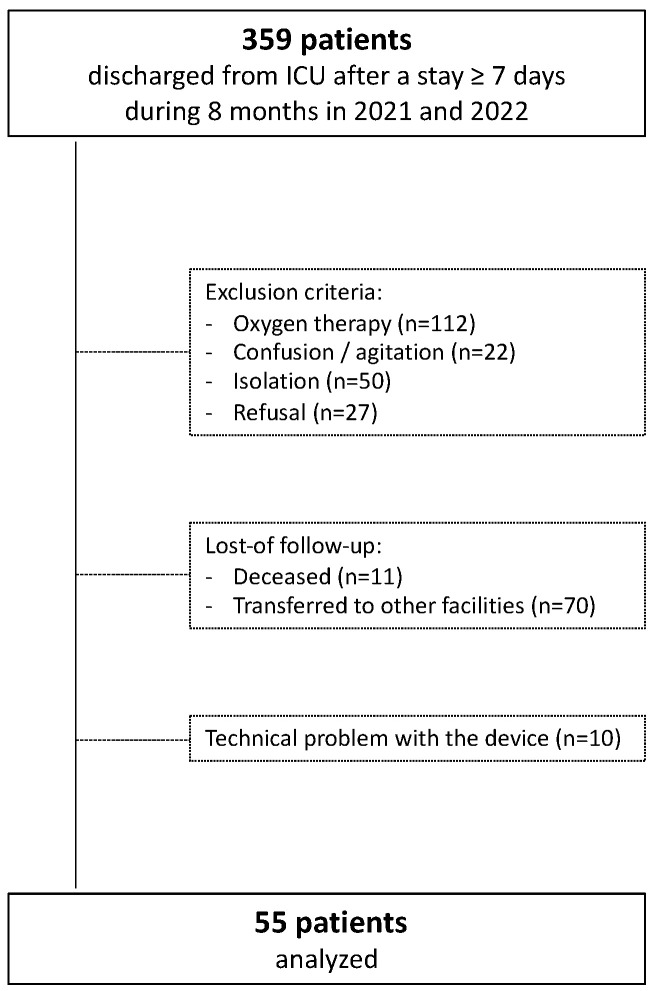
Flow chart.

During the 8 months of recruitment, 359 patients were discharged alive from the ICU after a stay ≥ 7 days. About one-third of these patients were on oxygen supply, precluding IC. Finally, a total of 55 patients were analyzed (Figure 1).

The characteristics of the patients are detailed in Table 2.

The anthropometric data are detailed in Table 3. Only 12 patients were obese (21.8%). At the time of inclusion, 4/55 (7.3%) were on intermittent hemodialysis. Most of the patients (31/55, 56.4%) were fed by the oral route.

Their biological data are detailed in Table 4. An inflammatory syndrome was observed in 49/55 (89.1%) patients. In patients with CRP in normal ranges, prealbumin was also in normal ranges. Albumin was below the normal range in 24/55 (43.6%) patients.

### 3.2. Indirect Calorimetry

The measurement was performed 4 (3–6) days after ICU discharge and lasted 17.5 (15–20) mins. The recorded data are detailed in Table 3. In women, the measured EE was 1478 (1199–1836) kcal/day, corresponding to 23.1 (20.9–24.4) kcal/kg/day. In men, the measured EE was 1762 (1415–2123) kcal/day, corresponding to 22.6 (18.6–24.4) kcal/kg/day.

### 3.3. Estimated Energy Expenditure

**Figure 2:**
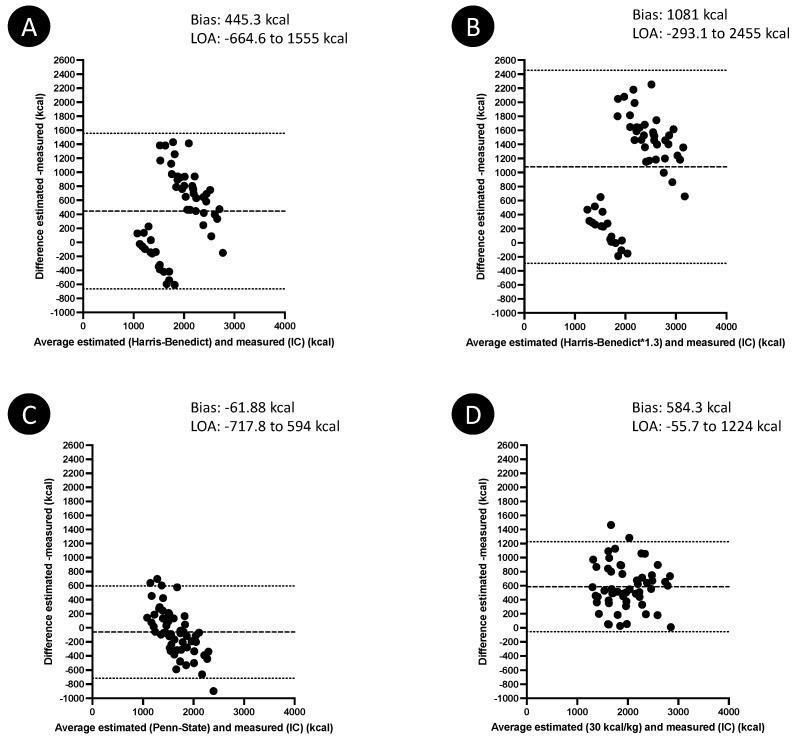
Bland–Altman plots showing the difference between estimated energy expenditure and measured energy expenditure using indirect calorimetry as the comparison method. The estimation was based on the Harris–Benedict equation (**A**), the Harris–Benedict equation corrected with a 1.3 stress factor (**B**), the Penn–State equation (**C**), and the 30 kcal/kg formula (**D**). Mean differences are represented by dashed lines, limits of agreement (LOA) are represented by dotted lines.

Estimated EE values are detailed in Table 3. The estimated EE using the Penn–State formula was similar to the measured EE (*p* = 0.145). On the contrary, the estimated EE values using the three other formulas were significantly higher than the measured EE (*p* < 0.001 for each test). All estimated EEs were significantly and positively correlated to the measured EE (*p* < 0.001 for each test): r_S_ = 0.59 (95% confidence interval (CI) 0.38–0.75) for the Harris–Benedict equations, r_s_ = 0.65 (95%CI 0.46–0.78) for the Penn–State equation, and r_s_ = 0.73 (95%CI 0.56–0.83) for the weight-based formula. The performances of the four predictive formulas are illustrated in Figure 2. A less significant bias between estimated and measured EE was observed with the Penn-State formula (bias −61.88 kcal). However, the agreement interval was large, with an underestimation of EE up to 717.8 kcal with the Penn-State equation compared to IC. The bias observed with the three other predictive formulas ranged from 445.3 to 1081 kcal (Figure 2).

Using the Penn–State equation, the predicted EE fell within 85% and 115% of the measured EE in 32/55 (58.2%) patients. The Penn–State equation led to an underestimation or an overestimation of EE in, respectively, 14/55 (25.4%) and 9/55 (16.4%) of the patients. With this equation, estimated EE represented −5.1% (−15.4–9.9%) of the measured EE.

## 4. Discussion

In the present cohort of survivors of a prolonged ICU stay, the resting EE measured by indirect calorimetry was around 22–23 kcal/kg/day during the week following ICU discharge, in a context of persistent low inflammation. This was significantly lower than the EE predicted by the 30 kcal/kg/day formula suggested by experts at this stage of the post-ICU trajectory [9]. Energy needs have been suggested to be higher during this period, partly in light of some publications reporting high EE during the third week following ICU admission [19]. The same observation has been reported more recently in COVID-19 patients: their measured EE reached 30 kcal/kg/day at similar timing [20]. However, these measurements were performed in patients still in the ICU, meaning indirect calorimetry was performed during a prolonged post-acute phase. In the present study, patients had obviously passed this phase, as they were discharged from the ICU. In a similar previously published study including a low number of survivors after ICU discharge [10], the measured EE did not reach 30 kcal/kg/day, but was rather close to 25 kcal/kg/day, as in our cohort. Whether the measured EE is equivalent to the actual energy needs in these patients is unclear. It has been suggested by experts that energy intakes should be increased to 125% of the measured EE in the post-ICU phase. However, it is not confirmed that such a strategy is associated with improved outcomes. Altogether, based on EE measurements, it seems that daily energy intakes should not be higher than 23–25 kcal/kg in the post-ICU hospitalization period, but this needs to be confirmed by further studies repeating IC measurements and analyzing outcomes in this category of patients.

In this study, the Penn–State equation was the only one to provide predicted EE statistically similar to the measured values, with a bias less than 100 kcal and an underestimation of about 5% compared to the measured EE. However, the performances of the Penn–State equations were weak: the limits of agreement in the Bland–Altman analysis were wide, and the predicted EE was found accurate in only 58% of the patients. This is not surprising, as several publications demonstrated that equations are generally inaccurate to predict EE in critically ill patients at almost all time points of their trajectory [21,22,23,24]. A reason for these inaccuracies is the complex and dynamic metabolic alterations characterizing the critical illness and the post-ICU condition. In addition, some external factors, such as medications, previous nutritional intakes or rehabilitation activity, are not taken into account in the equations. All these parameters also explain the huge inter-individual variability in measured energy expenditure observed in the present study, as well as in another similar cohort [10].

The metabolism variability between patients and the subsequent inaccuracy of predictive equations are arguments for the use of indirect calorimetry as frequently as possible, or at least when any significant change in clinical condition occurs. Concerns were raised about the past generation of indirect calorimeters in relation to their availability, their accuracy, their calibration and maintenance protocols, and their practical use [12,25]. Given the recent commercialization of a new generation of accurate and simple devices (the Q-NRG, Cosmed, Rome, Italy and Baxter, Deerfield, IL, USA), the implementation of an individualized nutritional care program is now possible and easier in daily practice [26]. Nutrition guided by indirect calorimetry during the ICU stay has been shown to be associated with reduced nutritional deficits and reduced catabolism [27], a lower incidence of ICU-acquired weakness [28] and decreased mortality [29]. This positive impact of indirect calorimetry needs to be confirmed in further large studies in ICU, but also in post-ICU settings. The appropriate outcomes for nutrition studies have recently been defined in a core outcome set [30].

In the present study, a validated and scrupulously calibrated device was used to measure EE in the post-ICU phase. However, some limitations need to be acknowledged. First, the cohort, despite being the largest studied so far, was limited. The external validity of the present results should be confirmed in other larger cohorts. Second, indirect calorimetry was not performed in a fasting condition. This could have biased the comparison between measured and predicted EE. Most of the equations predict the resting EE only, theoretically not including diet-induced thermogenesis [31]. The increase in metabolic rate due to diet varies according to the meal and whether overfeeding has occurred. Continuous feeding infusion induces limited thermogenesis. However, the critical care prediction equations are known to include diet-induced thermogenesis; no adjustment is required for the Penn–State equation or the Harris–Benedict corrected with a stress factor [32]. Third, only a single indirect calorimetry measurement was performed. It would be useful to repeat the measures during the post-ICU trajectory, as the physiology and needs can change during this period. Moreover, the ability to detect these changes is lacking, hence indirect calorimetry can help. Fourth, the predictive equations used in this study were empirically chosen, as no equations have been validated to date in the post-ICU context. This choice may have excluded other formulas that could have demonstrated better permeances, as the accuracy of equations may change over the course of an illness. Finally, nutritional intakes were not quantified. This should have allowed the calculation of the energy balance and the diagnosis of under- or over-nutrition.

## 5. Conclusions

In the present cohort of patients who survived a prolonged ICU stay, energy expenditure measured by indirect calorimetry was around 22–23 kcal/kg/day. During the post-ICU hospitalization, none of the equations performed accurately in predicting the energy expenditure measured by indirect calorimetry. Further studies should confirm these findings and determine whether indirect calorimetry-guided nutrition in the post-ICU phase could improve outcomes.

## Figures and Tables

**Table 1:** Estimated energy needs: predictive equations.

Equations	Estimated Resting Energy Expenditure
Harris–Benedict (HB), kcal/day	Men: (13.75 × W) + (5 × H) − (6.8 × age) + 66 Women: (9.6 × W) + (1.8 × H) − (4.7 × age) + 655
Penn–State (PS), kcal/day	*Mifflin:* Men: (10 × W) + (6.25 × H) − (5 × age) + 5 Women: (10 × W) + (6.25 × H) − (5 × age) − 161 *Penn-State:* PS = (Mifflin × 0.94) + (36.6 × 186) − 6597
Post-ICU, kcal/kg/day	30

H: height (cm); ICU: intensive care unit; W: weight (kg).

**Table 2:** Patients’ characteristics.

Data		Cohort *n* = 55	Men *n* = 37 (67.3%)	Women *n* = 18 (32.7%)
Age, y		60 (52–67)	58 (52–67.5)	64 (50.5–67.8)
Comorbidities, *n* (%)	Cardiovascular ^a^	38 (69.1)	26 (70.3)	12 (66.7)
	Respiratory ^b^	15 (27.3)	12 (32.4)	3 (16.7)
	Digestive ^c^	26 (47.3)	19 (51.4)	7 (22.2)
	Metabolic and endocrine ^d^	20 (36.4)	13 (35.1)	7 (22.2)
	Chronic kidney disease	28 (50.9)	22 (59.5)	6 (33.3)
	Oncologic	6 (11)	3 ((8.1)	3 (16.7)
	Chronic alcoholism	17 (30.9)	15 (40.5)	2 (11.1)
SAPS II		40 (25.7–54.5)	46 (24.7–64.3)	34 (24.5–45)
Admission reason, *n* (%)	Medical	30 (54.5)	17 (45.9)	13 (72.2)
	Surgical	25 (45.5)	20 (54.1)	5 (27.8)
Admission failure, *n* (%)	Cardiovascular	21 (38.2)	16 (43.2)	5 (27.8)
	Pulmonary	10 (18.2)	8 (21.6)	2 (11)
	Neurologic	12 (21.8)	3 (8.2)	9 (50)
	Digestive	5 (9)	4 (10.8)	1 (5.6)
	Trauma	3 (5.5)	2 (5.4)	1 (5.6)
	Other	4 (7.3)	4 (10.8)	0
Mechanical ventilation, *n* (%)		39 (70.9)	28 (75.7)	11 (61.1)
Duration of mechanical ventilation, days		6 (2–10)	5 (2–9)	7 (2–15)
Renal replacement therapy (RRT), *n* (%)		2 (3.6)	1 (2.7)	1 (5.6)
RRT duration, days		6 and 9	9	6
Insulin, *n* (%)		35 (63.6)	25 (67.6)	10 (55.6)
Insulin duration, days		6 (2–11)	6 (1.5–11.5)	6 (3.2–8.5)
Nutrition route, *n* (%)	PO	17 (30.9)	13 (35.2)	4 (22.2)
	PO + EN	6 (11)	4 (10.8)	2 (11)
	EN	24 (43.6)	14 (37.8)	10 (55.6)
	EN + PN	5 (9)	4 (10.8)	1 (5.6)
	PN	3 (5.5)	2 (5.4)	1 (5.6)
ICU LOS, days		12 (7–16)	12 (7–16)	11.5 (7–17)
Hospital LOS, days		25 (17–41)	22 (17–32.2)	34 (20–58)

Data are expressed as median with lower and upper quartiles (Q1-Q3). EN: enteral nutrition; ICU: intensive care unit; LOS: length of stay; PO: per os; PN: parenteral nutrition; SAPS II: Simplified Acute Physiology Score. ^a^ Ischemic heart disease, valvular disease, cardiomyopathies, chronic heart disease, hypertension. ^b^ Asthma, chronic obstructive pulmonary disease and interstitial lung diseases. ^c^ Gastro-esophageal reflux, ulcers, digestive surgery, pancreatitis. ^d^ Hypo- or hyperthyroidism, hyperlipidemia.

**Table 3:** Nutritional data.

Data		Cohort *n* = 55	Men *n* = 37 (67.3%)	Women *n* = 18 (32.7%)	*p* Value
Actual weight, kg		77 (63–94)	83.7 (64–96.8)	71 (58.2–81.7)	0.048
Actual weight considered for nutritional calculation, kg		74 (63–88)	80 (64–92.3)	70.8 (58.2–76.1)	0.011
BMI, kg/m^2^		26.1 (22.2–29.7)	26 (21.7–29.6)	26.6 (22.8–29.9)	0.528
Nutrition route, *n* (%)	PO	31 (56.4)	22 (59.4)	9 (50)	
	PO + EN	7 (12.7)	3 (8.2)	4 (22.2)	
	EN	15 (27.3)	10 (27)	5 (27.8)	
	PN	1 (1.8)	1 (2.7)	0	
	PO + PN	1 (1.8)	1 (2.7)	0	
VO_2_, mL/min		243.5 (188.5–281)	257 (202–302.5)	217 (170.5–257)	0.091
VCO_2_, mL/min		189.5 (155.3–238)	196.5 (157.8–245.8)	180 (135.8–202.3)	0.158
RQ		0.83 (0.76–0.88)	0.83 (0.77–0.89)	0.80 (0.72–0.88)	0.374
Measured EE, kcal/day		1682 (1328–1975)	1762 (1415–2123)	1478 (1199–1836)	0.093
Measured EE, kcal.kg/day		22.9 (19.1–24.2)	22.6 918.6–24.4)	23.1 (20.9–24.4)	0.756
Predicted EE, kcal/day	Harris–Benedict	2344 (1389–2563)	2500 (2335–2690)	1344 (1242–1394)	<0.001
	Harris–Benedict corrected with a stress factor of 1.3	3048 (1805–3332)	3250 (3036–3496)	1747 (1614–1813)	<0.001
	Penn–State	1589 (1443–1809)	1704 (1529–1888)	1385 (1285–1457)	<0.001
	30 kcal/kg	2220 (1890–2640)	2400 (1920–2769)	2124 (1748–2283)	0.011

Data are expressed as median with lower and upper quartiles (Q1–Q3). BMI: body mass index; EE: energy expenditure; EN: enteral nutrition; PO: per os; PN: parenteral nutrition; RQ: respiratory quotient; VO_2_: oxygen consumption; VCO_2_: carbon dioxide production.

**Table 4:** Biological parameters.

Blood Analysis	Reference Ranges	*n* = 55
C-reactive protein (CRP), mg/L	0–5	29.1 (11.4–60.8)
Total protein, g/L	58–83	60 (55–69.5)
Albumin, g/L	≤60 years: 35–52 >60 years: 32–46	32 (29.7–36)
Prealbumin, g/L	0.2–0.4	0.21 (0.18–0.27)

Data are expressed as median with lower and upper quartiles (Q1–Q3).

## Data Availability

The datasets used and/or analyzed during the current study are available from the corresponding author on reasonable request.

## References

[1] Preiser J.C., van Zanten A.R., Berger M.M., Biolo G., Casaer M.P., Doig G.S., Griffiths R.D., Heyland D.K., Hiesmayr M., Iapichino G. (2015). Metabolic and nutritional support of critically ill patients: Consensus and controversies. Crit. Care.

[2] Rougier L., Preiser J.C., Fadeur M., Verbrugge A.M., Paquot N., Ledoux D., Misset B., Rousseau A.F. (2020). Nutrition During Critical Care: An Audit on Actual Energy and Protein Intakes. J. Parenter. Enter. Nutr..

[3] Ridley E.J., Chapple L.S., Chapman M.J. (2020). Nutrition intake in the post-ICU hospitalization period. Curr. Opin. Clin. Nutr. Metab. Care.

[4] Moisey L.L., Pikul J., Keller H., Yeung C.Y.E., Rahman A., Heyland D.K., Mourtzakis M. (2021). Adequacy of Protein and Energy Intake in Critically Ill Adults Following Liberation From Mechanical Ventilation Is Dependent on Route of Nutrition Delivery. Nutr. Clin. Pract..

[5] Peterson S.J., Tsai A.A., Scala C.M., Sowa D.C., Sheean P.M., Braunschweig C.L. (2010). Adequacy of oral intake in critically ill patients 1 week after extubation. J. Am. Diet. Assoc..

[6] Merriweather J., Smith P., Walsh T. (2014). Nutritional rehabilitation after ICU—Does it happen: A qualitative interview and observational study. J. Clin. Nurs..

[7] Prado C.M., Landi F., Chew S.T., Atherton P.J., Molinger J., Ruck T., Gonzalez M.C. (2022). Advances in muscle health and nutrition: A toolkit for healthcare professionals. Clin. Nutr..

[8] Rousseau A.F., Prescott H.C., Brett S.J., Weiss B., Azoulay E., Creteur J., Latronico N., Hough C.L., Weber-Carstens S., Vincent J.L. (2021). Long-term outcomes after critical illness: Recent insights. Crit Care.

[9] Van Zanten A.R.H., De Waele E., Wischmeyer P.E. (2019). Nutrition therapy and critical illness: Practical guidance for the ICU, post-ICU, and long-term convalescence phases. Crit. Care.

[10] Ridley E.J., Parke R.L., Davies A.R., Bailey M., Hodgson C., Deane A.M., McGuinness S., Cooper D.J. (2019). What Happens to Nutrition Intake in the Post-Intensive Care Unit Hospitalization Period? An Observational Cohort Study in Critically Ill Adults. J. Parenter. Enter. Nutr..

[11] Singer P., Blaser A.R., Berger M.M., Alhazzani W., Calder P.C., Casaer M.P., Hiesmayr M., Mayer K., Montejo J.C., Pichard C. (2019). ESPEN guideline on clinical nutrition in the intensive care unit. Clin. Nutr..

[12] Wischmeyer P.E., Molinger J., Haines K. (2021). Point-Counterpoint: Indirect Calorimetry Is Essential for Optimal Nutrition Therapy in the Intensive Care Unit. Nutr. Clin. Pract..

[13] Oshima T., Delsoglio M., Dupertuis Y.M., Singer P., De Waele E., Veraar C., Heidegger C.P., Wernermann J., Wischmeyer P.E., Berger M.M. (2020). The clinical evaluation of the new indirect calorimeter developed by the ICALIC project. Clin. Nutr..

[14] Oshima T., Berger M.M., De Waele E., Guttormsen A.B., Heidegger C.P., Hiesmayr M., Singer P., Wernerman J., Pichard C. (2017). Indirect calorimetry in nutritional therapy. A position paper by the ICALIC study group. Clin. Nutr..

[15] Delsoglio M., Dupertuis Y.M., Oshima T., van der Plas M., Pichard C. (2020). Evaluation of the accuracy and precision of a new generation indirect calorimeter in canopy dilution mode. Clin. Nutr..

[16] Gandotra S., Files D.C., Shields K.L., Berry M., Bakhru R.N. (2021). Activity Levels in Survivors of the Intensive Care Unit. Phys. Ther..

[17] Fraipont V., Preiser J.C. (2013). Energy estimation and measurement in critically ill patients. J. Parenter. Enter. Nutr..

[18] Frankenfield D.C., Ashcraft C.M. (2017). Toward the Development of Predictive Equations for Resting Metabolic Rate in Acutely Ill Spontaneously Breathing Patients. J. Parenter. Enter. Nutr..

[19] Uehara M., Plank L.D., Hill G.L. (1999). Components of energy expenditure in patients with severe sepsis and major trauma: A basis for clinical care. Crit. Care Med..

[20] Whittle J., Molinger J., MacLeod D., Haines K., Wischmeyer P.E., Group L.-C.S. (2020). Persistent hypermetabolism and longitudinal energy expenditure in critically ill patients with COVID-19. Crit. Care.

[21] Zusman O., Kagan I., Bendavid I., Theilla M., Cohen J., Singer P. (2019). Predictive equations versus measured energy expenditure by indirect calorimetry: A retrospective validation. Clin. Nutr..

[22] Vasileiou G., Qian S., Iyengar R., Mulder M.B., Gass L.M., Parks J., Pust G.D., Rattan R., Lineen E., Byers P. (2020). Use of Predictive Equations for Energy Prescription Results in Inaccurate Estimation in Trauma Patients. Nutr. Clin. Pract..

[23] Smetana K.S., Hannawi Y., May C.C. (2021). Indirect Calorimetry Measurements Compared With Guideline Weight-Based Energy Calculations in Critically Ill Stroke Patients. J. Parenter. Enter. Nutr..

[24] Israfilov E., Kir S. (2021). Comparison of Energy Expenditure in Mechanically Ventilated Septic Shock Patients in Acute and Recovery Periods via Indirect Calorimetry. J. Parenter. Enter. Nutr..

[25] De Waele E., Honore P.M., Spapen H.D. (2016). New generation indirect calorimeters for measuring energy expenditure in the critically ill: A rampant or reticent revolution?. Crit. Care.

[26] De Waele E., Jonckheer J., Wischmeyer P.E. (2021). Indirect calorimetry in critical illness: A new standard of care?. Curr. Opin. Crit. Care.

[27] Gonzalez-Granda A., Seethaler B., Haap M., Riessen R., Bischoff S.C. (2021). Effect of an intensified individual nutrition therapy on serum metabolites in critically ill patients—A targeted metabolomics analysis of the ONCA study. Clin. Nutr. ESPEN.

[28] Fetterplace K., Beach L.J., MacIsaac C., Presneill J., Edbrooke L., Parry S.M., Rechnitzer T., Curtis R., Berney S., Deane A.M. (2019). Associations between nutritional energy delivery, bioimpedance spectroscopy and functional outcomes in survivors of critical illness. J. Hum. Nutr. Diet..

[29] Duan J.Y., Zheng W.H., Zhou H., Xu Y., Huang H.B. (2021). Energy delivery guided by indirect calorimetry in critically ill patients: A systematic review and meta-analysis. Crit. Care.

[30] Davies T.W., van Gassel R.J.J., van de Poll M., Gunst J., Casaer M.P., Christopher K.B., Preiser J.C., Hill A., Gundogan K., Reintam-Blaser A. (2022). Core outcome measures for clinical effectiveness trials of nutritional and metabolic interventions in critical illness: An international modified Delphi consensus study evaluation (CONCISE). Crit. Care.

[31] Achamrah N., Delsoglio M., De Waele E., Berger M.M., Pichard C. (2021). Indirect calorimetry: The 6 main issues. Clin. Nutr..

[32] Frankenfield D.C., Ashcraft C.M. (2011). Estimating energy needs in nutrition support patients. J. Parenter. Enter. Nutr..

